# Synthesis, characterization, and exosomal corona formation of self-assembled dipeptide nanomaterials

**DOI:** 10.1038/s41598-025-98706-5

**Published:** 2025-04-19

**Authors:** Burcu Önal Acet, Ömür Acet, Madita Wandrey, Roland H. Stauber, Désirée Gül, Mehmet Odabaşı

**Affiliations:** 1 Faculty of Arts and Science, Chemistry Department, Biochemistry Division, Aksaray University, Aksaray, 68100 Turkey; 2 Department of Otorhinolaryngology Head and Neck Surgery, Molecular and Cellular Oncology, University Medical Center, Mainz/ENT Building 102, Langenbeckstraße 1, Mainz, 55131 Germany; 3 Vocational School of Health Science, Pharmacy Services Program, Tarsus University, Tarsus, Turkey

**Keywords:** Extracellular vesicles, Exosome, Fmoc-Lysin nanomaterials, Dipeptides, Biomolecule corona, Exosomal corona, Structural biology, Biochemistry, Biological techniques, Biophysics, Cancer, Chemistry, Materials science

## Abstract

Exosomes (Exos), also known as small extracellular vesicles, are naturally occurring nanoparticles (NPs), which are characterized by their nanometer size and negative charged in physiological environments. While it is widely accepted that proteins and biological compounds adhere to different nanomaterials (NMs), forming an outer layer known as the biomolecule corona (BC), the detailed understanding of factors contributing to BC formation as well as of its biological effects remains limited. Studies have shown that BC formation can affect the physicochemical properties of synthetic and natural NPs once contacting biological fluids. Here, we present a study investigating the novel concept of exosomal corona formation, which in contrast to the well-documented BC mainly consists of Exos/exosomal components. For this purpose, peptide-based Fmoc-Lysine (Fmoc-Lys) NMs were synthesized and characterized, and interaction studies with (cancer) cell-derived Exos were performed. Measurements of size, zeta potential, and colloidal stability indicate exosomal corona formation. Furthermore, cell viability experiments showed that the Exo-NM interaction resulted in reduced nanotoxicity profile indicating practical relevance for biological applications of these NMs. In summary, here we provide first evidence supporting the concept of exosomal corona formation around NMs that should become part of evaluating interactions at nano-bio-interfaces.

**Supplementary Information:**

The online version contains supplementary material available at 10.1038/s41598-025-98706-5.

## Introduction

Recent advances in the field of nanotechnology have made it possible to obtain new highly functional materials for biomedical applications^1–4^. As size decreases to the nanoscale, many unique optical, magnetic and electrical properties emerge that make nanomaterials different from traditional macromolecules^5^. The selection and design of nanostructures and the tuning of their size and surface properties are extremely important, especially for precision medicine-based applications and targeted drug delivery^3^. Here, peptide-based NMs offer an advantageous approach due to their versatile structure and function, as well as an excellent thermal and mechanical stability^6^. Synthesis can be achieved by straightforward self-assembly of individual peptides. Furthermore, peptide-based self-assembled NMs seem particularly suitable for medical applications due to their biological activity and excellent biocompatibility^7^. The process of self-assembly does not only confer higher stability and mechanical strength to protein and peptide structures, but further enhances their inherent activity and function^8^.

Especially nanoparticular dipeptides, which were discovered through self-assembly synthesis, are of great interest for the biomedical field. Recently, these materials have demonstrated their biological compatibility, low-cost and ease of synthesis, functionality, tuneability, and nanodimensions^6^. Due to the limited number of naturally occurring amino acids, self-assembly with modified amino acids has been of great interest mostly focusing on 9-fluorenylmethoxycarbonyl (Fmoc)-terminated materials^9–12^. With its inherent hydrophobic and aromatic nature, Fmoc-based modification of amino acids results in NMs exhibiting relatively fast self-assembly kinetics, excellent physical and chemical properties, and great potential in cell culture, photocatalysis, drug delivery and antibacterial applications^13^. Recently, extensive characterization of self-assembled Fmoc-Lysine based structures in different organic solvents have been reported^14^. This structure may pave the way for the designs of a new class of self-assembled NMs synthesized to fulfill desired biomedical functions.

Recent studies have revealed that protein complexes around Exos are driven primarily by electrostatic interactions and protein aggregation in aqueous environments^15^. However, our understanding of the factors influencing the formation of these protein complexes in biofluids remains in its early stages, lacking both robust theoretical models and experimental validation^15–17^.

The biomedical application of NMs including self-assembled dipeptide NMs always comes along with several challenges of the biological environment. It is widely accepted that NMs quickly acquire layers of biomolecules upon contact with biological fluids, the so-called biomolecule corona (BC). Here, the binding of proteins in the form of a protein corona (PC) is well known for different types of NMs^18^. The formation of the BC significantly influences not only the stability and distribution of NMs in biological systems but also their interactions with cells and tissues. It is crucial to understand that this dynamic process can modulate the therapeutic efficacy of NMs, potentially altering their intended functions. Consequently, a deeper insight into the mechanisms by which biomolecules interact with NMs will pave the way for optimizing their design, ensuring that they achieve desired outcomes in drug delivery, imaging, and targeted therapies^19^.

Importantly, extracellular environments contain not only soluble proteins and other “free” biomolecules, but also complex systems of macromolecules, such as the extracellular matrix, as well as small vesicles representing extracellular compartments of their origin cells. Extracellular vesicles (EVs) are membrane-coated particles, which play a critical role in intercellular communication. In the past decade, EVs have been the focus of extensive research due to their involvement in various physiological and pathological processes. Based on their size, origin, and biogenesis, EVs can be classified into exosomes (small EVs, 30–150 nm), microvesicles (100 nm–1 μm), and apoptotic bodies (1–5 μm)^20^. Exosomes (Exos) are of particular interest as naturally occurring nanoparticles with characteristic endosomal biogenesis and specific biomolecular cargoes. Furthermore, Exos, being released by a wide range of cell types, are present in large quantities in biological fluids, where they interact with biomolecules and applied therapeutics, such as nanoparticular drugs. Whereas there are previous studies showing BC formation around Exos in aqueous environments^15^, the novel concept of an “exosomal corona” has received little attention in the literature. We define the exosomal corona as a BC mainly consisting of intact exosomes or exosomal components and/or cargo. This has to be clearly distinguished from the well-documented protein corona, since the exosomal corona can include a complex mixture of biomolecules, not only proteins, but also lipids and nucleic acids.

Here, we synthesized Fmoc-Lys dipeptide-based NMs for the use in interaction studies with Exos. After the synthesis stage, characterization studies using SEM, TEM, DLS and ATR-FTIR were performed. We carried out interaction studies of synthesized Fmoc-Lys NMs with cell-derived Exos using different physiological conditions. We found, for the first time to our knowledge, that Exos form biomolecular corona-like structures around Fmoc-Lys dipeptides indicated by changes in size and zeta potential. To analyze potential effects of exosomal corona formation, we measured nanotoxicity profiles of Fmoc-Lys NMs and control amorphous silica nanoparticles (SiNPs) in head and neck cancer (HNC) cell lines. Here, we confirmed that interaction with Exos could alter nanotoxicity profiles of analyzed NMs. The results indicate a practical relevance of exosomal corona formation around NMs, which could be exploited to attain a significant reduction of nanotoxicity.

## Materials and methods

### Nanoparticles and chemicals

AmSil 20 amorphous silica nanoparticles were purchased from Nyacol, USA. Characterization of AmSil 20 has been previously reported^18^. Unless stated otherwise, chemicals were purchased from Sigma Aldrich/Merck (Darmstadt, Germany). Cell culture media and reagents were sourced from Gibco/Thermo Fisher Scientific (Dreieich, Germany). Consumables were purchased from Greiner Bio-One (Frickenhausen, Germany). Water used in experiments was purified by using a Barnstead (Dubuque, IA, USA) ROpure LP^®^ reverse osmosis unit.

### Synthesis of Fmoc-Lys based nanomaterials

Covalent cross-linked molecules based on Schiff base (C = N) between the amine group of the *N*-(fluorenylmethoxycarbonyl)-L-lysine molecule and the aldehyde group of the glutaraldehyde molecule used as a cross-linker are self-assembled to form Fmoc-Lys-NMs. Therefore, a 0.1 g/mL stock solution was prepared by dissolving Fmoc-Lys in 1,1,1,3,3,3-hexafluoro-2-propanol (HFIP). 0.9 mL of 25% glutaraldehyde (GA) solution was added to this stock solution. Prepared solution was taken into a vial and 2.1 mL of pure water was added to reduce the activity of GA, and incubated in the dark overnight to complete the self-assembly process. Formed nanomaterials were centrifuged at 20.000 rpm for 30 min, and washed 3 times with deionized water to eliminate remaining HFIP and GA. The resulting Fmoc-Lys NMs were dispersed in 3 mL of pure water, and stored at 4 °C for characterization studies.

### Characterization studies

For the detailed characterization of synthesized Fmoc-Lys NMs were performed by SEM, TEM, DLS and ATR-FTIR analyses. SEM imaging of Fmoc-Lys NMs was carried out using Carl Zeiss EVO 50 EP. 5 µL of NMs dispersion was dropped onto the clean glass surface, dried under nitrogen gas, and prepared for SEM analysis by gold coating. TEM analysis was performed using JEOL JEM-1230 at an accelerating voltage of 100 kV. The average diameter of Fmoc-Lys NMs was calculated by the size distribution analysis graph made with ImageJ application (https://imagej.nih.gov/ij/).

The average diameter and polydispersity index (PDI) of Fmoc-Lys NMs were determined by the equation given below^21^.

$$\:PDI={(Standart\:deviation/mean\:particle)}^{2}$$

In this equation, the values ​​between 0 and 0.1 and 0.1–1 indicate monodispersity and polydispersity, respectively.

Determination of the hydrodynamic diameters and zeta potential of Fmoc-Lys NMs, Exos, and Fmoc-Lys-Exo complexes were performed by DLS measurements at different pH and concentrations using the Nano Zetasizer device (NanoS, Malvern Instruments, London, UK). Results shown are the average of five independent measurements.

ATR-FTIR (Perkin Elmer, 400, FTIR spectrophotometer) analyzes were performed to demonstrate the synthesis of Fmoc-Lys NMs through functional groups. The FTIR spectra were utilized to determine the overall light reflection from the surface within the wavelength range of 650–4000 cm^−1^ with a resolution of 2 cm^−1^.

### Interaction assay

For determination of the optimal interaction rates also stated in Table 1, Fmoc-Lys NMs (0.5 or 1 mg/mL), SiNPs at toxic values ​​(25–60 µg/mL) and isolated Exos (0.2 µg/mL) were incubated at physiological pH range (pH 6.5, 7.0, 7.4). All interaction reactions were performed in PBS buffer with gentle rotation at 10 rpm for 3 h. A rotator system (Stuart^®^ SB3) was used for all interaction experiments to ensure even mixing of the samples. Following incubation, size and zeta potential analysis of pristine Fmoc-Lys NMs, SiNPs, pure exosomes, as well as co-incubated samples were performed. Finally, the maximum interaction rate was calculated.

### Colloidal stability

The quantification of colloidal stability of nanostructures can be effectively measured only in aqueous solutions that contain simple electrolytes^22^. For this purpose, Exos, Fmoc-Lys NMs-Exo and SiNPs-Exo structures were dissolved in PBS buffer (4 °C, pH 6.5, 72 h) to test the stability and final charges and sizes of the structures. At the end of 72 h, ZP and ZS values were measured, and the results were recorded.

### Cell culture

For Exos isolation and cell viability measurements, the established head and neck cancer cell line FaDu and HeLa cells were used. Thawed cells were regularly monitored by visual inspection and were used within a maximum of 20 passages for all experiments. Cell line authentication was confirmed at reasonable intervals by short tandem repeat (STR) profiling. FaDu cells were cultured in Dulbecco’s Modified Eagle’s Medium, and fetal bovine serum (FBS, 10%), 1% glutamine, and 1% penicillin–streptomycin as described before [27]. HeLa cells (CCL-2 ATCC) were grown in full DMEM with stable glutamine, 4.5 g/L glucose (Life Technologies, Paisley, U.K.), 10% FBS (Life Technologies, Paisley, U.K.), 100 U/mL penicillin, and 0.1 mg/mL streptomycin (Millipore-Sigma, Burlington, MA, United States) at 37 °C in a humidified air atmosphere with 5% CO_2_.

### Exosome isolation

Isolation of Exos was carried out by differential centrifugation as described before^23^. To collect cell supernatant, (FaDu/HeLa) cells were cultured in at least four 145 mm cell culture dishes to 80–90% confluency. To remove exosomal contamination from serum, cells were incubated in serum-free media for at least 48 h. Cell supernatant was collected and used for differential centrifugation. Centrifugation was performed using ultracentrifuge (Beckman Coulter, Avanti JXN-30 Centrifuge, USA). To remove cells, supernatant was centrifuged at 500 x g for 10 min at 4 °C. The supernatant was then collected and filtered through 0.22 mm filters (Merck Millipore) to remove contaminating apoptotic bodies, microparticles, and cell debris. Flow-through media was centrifugated at 100.000 x g, 4 °C for 90 min with a swing-out rotor JS24.38 to pellet Exos. The supernatant was carefully removed, and pellets containing crude Exos were resuspended and pooled in 1 mL of ice-cold PBS. A second round of ultracentrifugation (100.000 x g, 4 °C for 90 min) was performed, and the resulting Exos pellets were resuspended in 500 µL of PBS. Characterization of isolated particles was performed according to MISEV guidelines including cryo-TEM, measurement of size distribution, and analysis of exosomal markers by immunoblotting (Suppl. Fig. S1).

### Cell viability

To measure cell viability Alamar Blue assays were performed as described before^24^. FaDu or Hela cells were seeded in 96-well plates (5000 cells/well, triplicates) and treatment with NPs was performed for 24 h after seeding at the indicated concentrations (*n* = 3). After different treatment time points (4, 24, 48 h), cell viability was assessed using Alamar Blue solution according to the manufacturer’s instructions. The medium was replaced with fresh medium containing 10% Alamar Blue and incubated for 1–2 h. Fluorescence was detected at 550 nm excitation and 595 nm emission wavelengths on a Tecan Spark^®^ (Tecan Group Ltd., Männedorf, Switzerland). Values of medium with Alamar Blue but without cells were subtracted and viability was normalized to untreated control samples incubated under the same conditions.

### Statistical analysis

An unpaired t-test was conducted for experiments that reported p-values. Unless specified otherwise, the p-values were derived from three independent experiments conducted in triplicates. Significance was determined based on p-values < 0.05 (*p < 0.05; **p < 0.01; ***p < 0.001, ****p < 0.0001). Statistical analysis and graph setup were carried out using Prism9 (Graph Pad Software).

## Results and discussion

### Characterization of Fmoc-Lysine NMs and exosomes

**Fig. 1:**
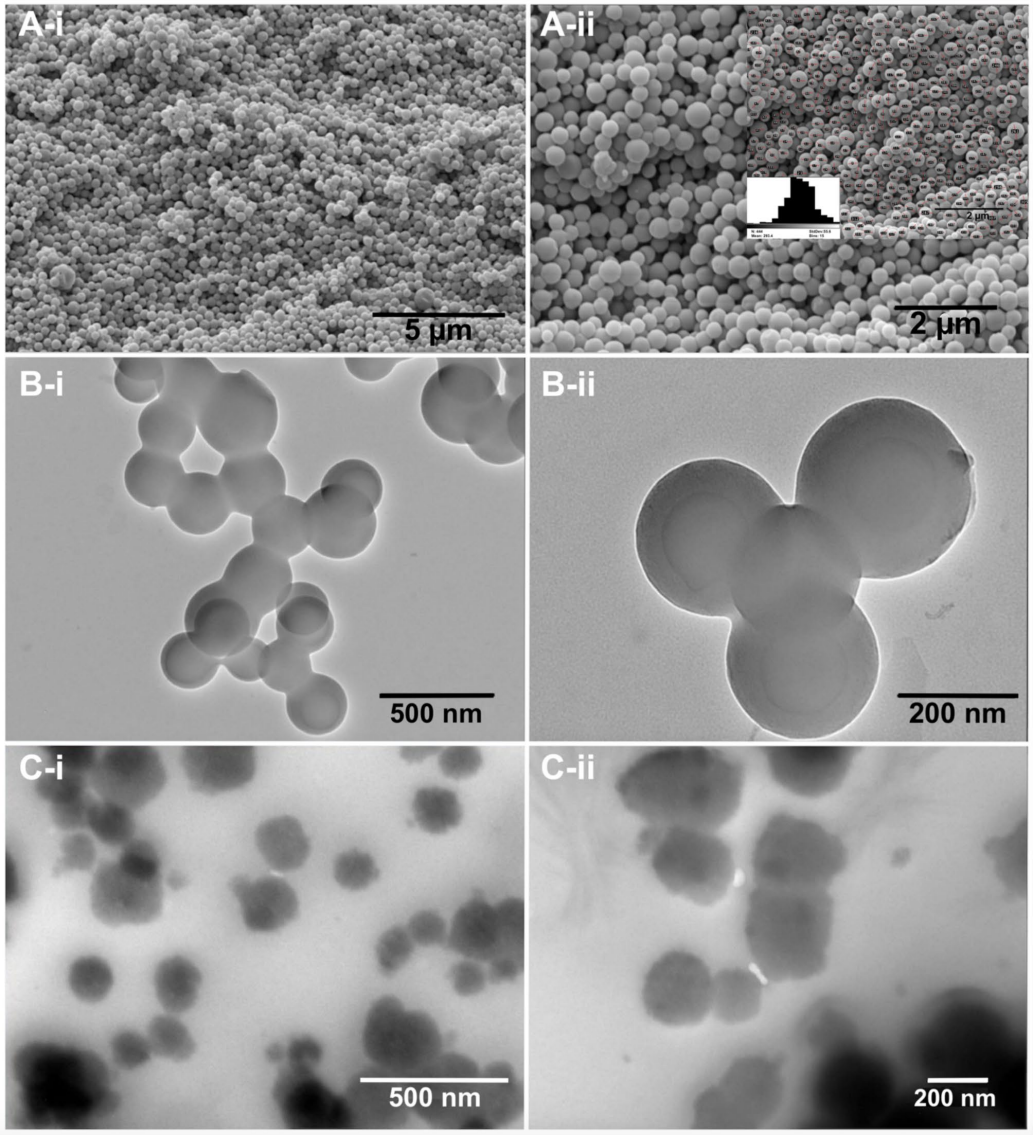
Characterization of Fmoc-Lys NMs. SEM (**A**) and TEM (**B**) images of pristine Fmoc-Lys NMs show uniform round shape and size. Panel A-ii contains results of SEM-based size distribution analysis. (**C**) TEM images of Fmoc-Lys NMs after incubation with isolated exosomes indicate increase of diameter and formation of a biomolecule corona.

After synthesis of Fmoc-Lys NMs, we performed state-of-the-art characterization studies. Acquisition and analysis of SEM, and TEM images revealed NMs of uniform round shape and size (Fig. 1A, B). The average diameter and polydispersity index (PDI) of Fmoc-Lys NMs were determined by the equation given in the methods section. According to data obtained from SEM image (*n* = 440 particles), average diameter and PDI of Fmoc-Lys NMs was calculated as 249.39 ± 47.21 nm and 0.0358, respectively. When compared to average diameter measured from DLS in Table 1(i.e., 293.4 ± 55.65), It is seen that the hydrodynamic size measured in the aqueous medium is larger than the size measured from the SEM photographs in the dry state. This situation, which is caused by the solvation of the particle in the aqueous medium, is important because it reflects the behavior of NMs in the aqueous medium^25^. According to guidelines shown in previous work, the nanostructures could be characterized as monodisperse^21^. Importantly, the morphology and size of Fmoc-Lys NMs obtained by SEM imaging seem to be comparable with results from TEM images (Fig. 1B).

Characterization of isolated extracellular vesicles was performed according to MISEV guidelines including cryo-TEM, measurement of size distribution, and analysis of exosomal markers by immunoblotting (Suppl. Fig. S1). Regarding the obtained particle size between 40 and 150 nm, isolated vesicles could be characterized as exosomes (Exos). After incubation of Fmoc-Lys NMs with isolated Exos, TEM imaging was performed (Fig. 1C). Here, we observed an increase of NMs diameter. Additionally, Fmoc-Lys NMs were surrounded by a cloudy structure due to the biological fluid effect. It is also indicative of a corona structure creating an unequal and non-versatile network surrounding the Fmoc-Lys NMs.

**Fig. 2:**
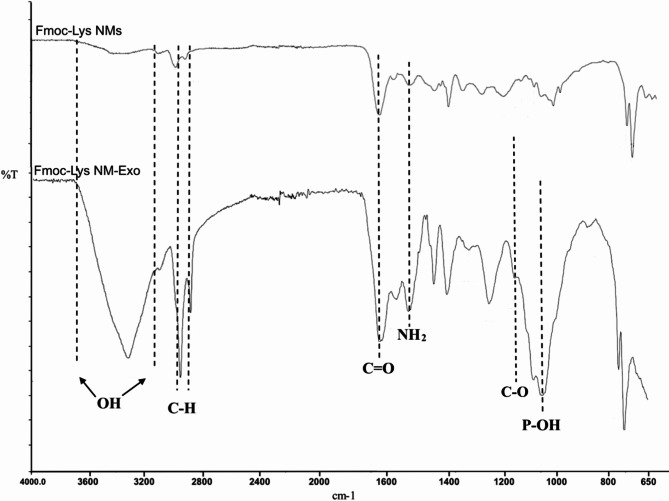
Spectra of pristine Fmoc-Lys NMs (top), and Fmoc-Lys NMs after incubation with Exos (below) obtained by ATR-FTIR analysis.

Next, ATR-FTIR analyses were performed to characterize the molecular composition of pristine Fmoc-Lys NMs and Fmoc-Lys NMs after incubation with Exos (Fig. 2).

The peaks at 1525 cm^−1^ for both spectra were ascribed to the N-H bending of the primary amine groups. While the absorption bands around 1630 cm^−1^ were attributed to the C = O stretching of the amide groups, the peaks around 1590 cm^−1^ were referred to the NH_2_ bending of the primary amine group for both structures. While the absorption bands at 2950 and 2890 cm^−1^for both spectra were referred to the C-H asymmetric and symmetric stretching^26^ of GA used as cross linker during the self-assembly process, increased peak intensities in the spectrum of Fmoc-Lys NMs-Exo referred to CH₂ symmetric and asymmetric stretching of lipids in Exos. Stretching peaks around 1050 and 1150 cm^−1^ were assigned to P-OH, and C-O groups of carbohydrates in Exo structures, respectively. The characteristic stretching vibration bands of hydrogen bonded hydroxyl groups in Exo structure are also seen around 3300 cm^−1^in the spectrum. In summary, the results of the ATR-FTIR analysis support that an exosomal corona structure is formed on Fmoc-Lys NMs^27^.

### Interaction of Fmoc-Lys NMs and exosomes

Fmoc-Lys NPs were selected, synthesized and characterized because they are functional and unique nanostructures as small as dipeptides. In this step, interaction studies of Fmoc-Lys NMs and exosomes were performed. Additionally, we included analysis of amorphous silica NPs (SiNPs) in our study serving as a reliable control with well-characterized physicochemical properties and a profiled protein corona^18,28^. Interaction studies were conducted with varying pHs and NP concentrations to mimic differing conditions in biological fluids. After 24 h incubation at 37 °C, zeta potential (ZP) and zeta size (ZS) of respective NPs and Exos was measured (Table 1), and diagrams were given in Fig. S2 (Supplementary File).

**Table 1:** Zeta potential and size values of pristine Fmoc-Lys NM, silica nanoparticles (SiNPs), pure exosomes (Exos), and co-incubated samples.

Sample	pH	Zeta potential (mV)	Zeta size (nm)	PDI
Fmoc-Lys NM	7.0	+ 19.40 ± 1.7	293.4 ± 55.65	0.103
SiNP		−35.80 ± 1.3	78 nm ± 5.3	0.155
Exos		−13.73 ± 2.1	101.1 nm ± 8.5	0.429
Fmoc-Lys NM (0.5 mg/mL)- Exos	6.5	−6.05 ± 1.4	329.4 nm ± 15.4	0.244
	7.0	−7.52 ± 1.3	315.3 nm ± 13.8	0.216
	7.4	−8.12 ± 1.4	306.8 nm ± 13.5	0.210
Fmoc-Lys NM (1 mg/mL)- Exos	6.5	−6.51 ± 1.8	311.7 nm ± 13.9	0.225
	7.0	−6.68 ± 1.7	306.1 nm ± 12.4	0.217
	7.4	−8.89 ± 1.1	301.2 nm ± 12.5	0.203
SiNP (25 µg/mL)-Exos	6.5	−11.42 ± 1.5	193.8 nm ± 11.3	0.166
	7.0	−16.04 ± 2.4	186.5 nm ± 8.5	0.172
	7.4	−26.78 ± 2.2	143.1 nm ± 8.4	0.169
SiNP (60 µg/mL)-Exos	6.5	−16.79 ± 1.9	173.5 nm ± 8.7	0.162
	7.0	−23.12 ± 2.3	139.7 nm ± 7.3	0.165
	7.4	−26.46 ± 1.8	148.4 nm ± 8.2	0.159

Initial ZP of Fmoc-Lys NMs, SiNP and Exos at pH 7.0 were found to be + 19.4, −35.8and − 3.73 mV. Interestingly, after co-incubation with Exos, positive ZPs of Fmoc-Lys NMs changed significantly into negative overall ZP in all analyzed samples. These findings suggest that Fmoc-Lys NMs are able to interact with Exos which may contribute to the formation of a biomolecule corona around the NM. The interaction is most likely based on electrostatic attraction that naturally occur between two molecules with opposite charges. Moreover, while the ZS value of pristine Fmoc-Lys NMs was determined with 293.4 nm, co-incubation with Exos increased Fmoc-Lys dimensions, with highest ZS measured at pH 6.5 (329.4 and 311.7 nm). Based on the observed results, we hypothesize that interaction of Exos with Fmoc-Lys NM increases at acidic pH. This observation could be relevant for an application in the tumor microenvironment for which acidification in similar pH ranges have been described^29^.

In comparison to the positively charged Fmoc-Lys NMs, interaction studies were also carried out with SiNPs representing well-characterized model NPs with negative charge^18^. Interestingly, measured ZP values of SiNPs became more positive after incubation with Exos, but still exhibit overall negativity. Here, there was no change in the effective attractive or repulsive forces between neighboring particles, and no aggregation was observed after interaction with Exos. Similar to the Fmoc-Lys NMs, largest Exo layers appear at pH 6.5. Based on these results, subsequent experiments were performed at pH 6.5 to induce strongest interactions.

In summary, our results indicate that Fmoc-Lys NMs form an Exo biomolecule corona, the exosomal corona based on electrostatic interaction. We hypothesize that intermolecular forces have a serious effect on the exosomal corona. For both NMs, Fmoc-Lys and SiNPs, we observed for the majority of the analyzed samples that the exosomal corona structure decreased slightly with increasing NP concentration. This phenomenon suggests that low concentrations of NPs in the medium are sufficient for the acquisition of maximal coronal thickness.

### Colloidal stability

Aggregation of NPs can have significant consequences, which can skew results and impede the reproducibility of experiments. This may primarily alter cellular uptake and affect the toxicity profile of the particles^22^. The attractive van der Waals forces arise from the interactions between induced, momentary, or permanent dipoles within the interatomic bonds of NPs and lead to the destabilization of colloidal system. Conversely, the electrostatic double layer of the NPs repels these attractive Van der Waals forces, thereby ensuring the stabilization of the dispersion. In aqueous environments, most NPs acquire a certain surface charge as a result of the ionization or dissociation of surface groups, or the adsorption of charged molecules or ions onto the particle surface. To maintain overall charge neutrality, a cloud of counterions forms around the particle^22,30,31^.

The colloidal stability of NPs is essential for effective handling, prolonged storage durations, and extended periods of cell incubation and circulation within the bloodstream. The magnitude of the zeta potential (ZP) can also give an indication of a particle´s stability in a colloid system. Whereas a large negative or positive ZP of particles in suspension can result in attraction or repulsion, low ZP values cannot prevent their interaction and may results in precipitation.

Zeta potential (ZP) values ​​also provide information about the stability of a particle in a colloidal system, but the evaluation of the interactions of NPs in biological environments can be significantly hindered by the aggregation of NPs in the environment they are in^32^. Therefore, it is still important to examine the stabilization behavior of NPs in biological environments and to evaluate possible corona formation results.

Measurements of ZP and ZS values after long-term (72 h) co-incubation of Fmoc-Lys NMs or SiNPs with Exos showed that the electrostatically interacting Fmoc-Lys-Exo complexes were more stable than the relatively weakly interacting SiNP-Exo structure (Table 2). In other words, it can be attributed to the fact that an electrostatic attraction force occurs between opposite poles and as a result, a more stable structure naturally occurs. Interestingly, an increase in the size of Exos was also observed after 72 h of incubation, probably because of their aggregation.

**Table 2:** Zeta potential and zeta size values for colloidal stability.

Sample	pH	Zeta potential (mV)	Zeta size (nm)
Exos (0.2 µg/mL)	6.5	−23.12	429.4 nm ± 1.4
Fmoc-Lys (0.5 mg/mL)-Exos	6.5	−6.19	351 nm ± 1.2
SiNP (25 µg/mL)- Exos	6.5	−13.4	143.1 nm ± 1.5

### Cell viability

Upon introduction into biological body fluids, nanomaterials rapidly bind biomolecules, such as proteins, lipids and other substances resulting in changes of nano-biological interactions^16^. Whereas protein corona formation on exosomes has been studied before^15^, the potential role of a biomolecule corona consisting of exosomes or exosomal components, the exosomal corona, represents a novel concept which is not well understood. Our shown results strongly indicate that Exos adsorb on the surface of analyzed NPs, and thus may provide novel biological identity affecting the interaction between NPs and cells.

**Fig. 3:**
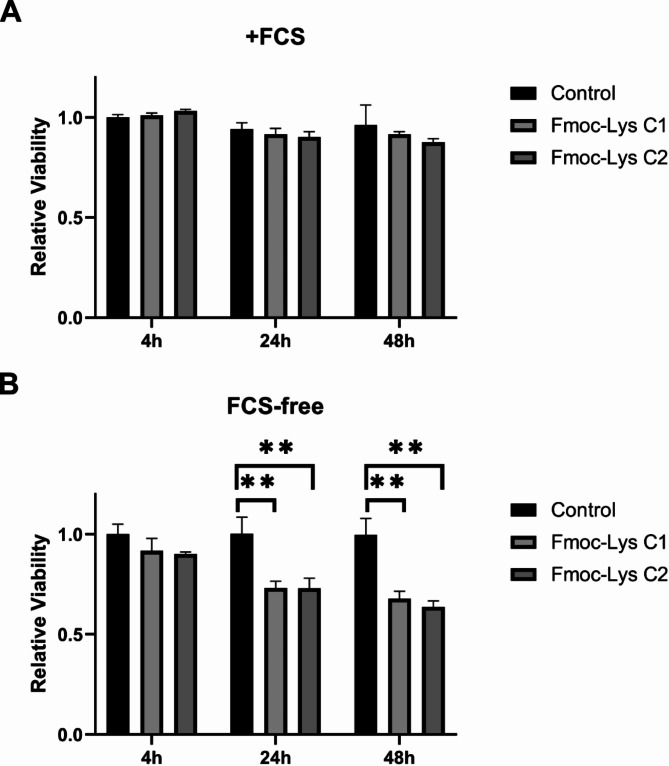
The presence of a biomolecule corona affects the toxicity profile of Fmoc-Lys NM. Cell viability tests were performed with FaDu cells treated with Fmoc-Lys NMs at different concentrations (C_1_: 0.5 mg/mL; C_2_: 1 mg/mL) in FCS + medium (**A**) and FCS free medium (**B**). Column, mean; bars, ±S.D., **p < 0.01. Columns shown without p-values were not significantly different.

To probe potential effects of a biomolecule corona formed on NMs, we performed cell viability analyses (Fig. 3). Therefore, the established head and neck cancer cell line FaDu was treated with Fmoc-Lys NPs (0.5 mg/mL; 1 mg/mL) in presence and absence of serum (Fig. 3). First, our study proved high biocompatibility of Fmoc-Lys NMs shown by low cellular toxicity in presence of serum, and thus after acquiring a biomolecule corona (Fig. 3A). In contrast, serum deprivation resulted in significantly reduced cell viability after Fmoc-Lys NMs treatment for at least 24 h (Fig. 3B). In a serum environment, NPs will acquire a biomolecule corona, which typically reduces the toxicity of NPs as we already have shown for other NMs, such as silica NPs^28^.

**Fig. 4:**
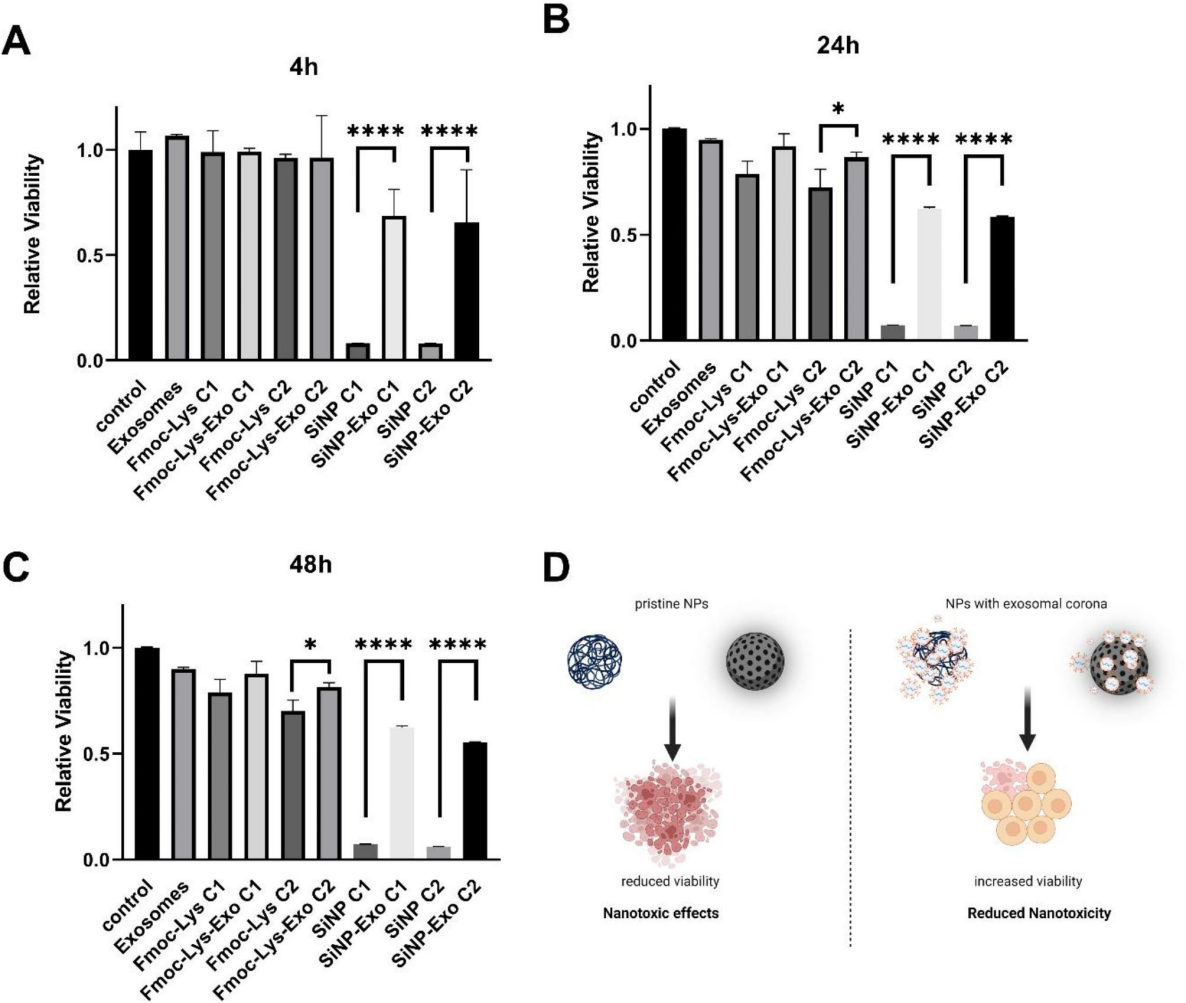
Acquisition of an exosomal corona on NPs is able to reduce nanotoxicity. (**A**–**C**) FaDu cells were treated with Fmoc-Lys NMs (C_1_: 0.5 mg/mL; C_2_: 1 mg/mL), Fmoc-Lys NMs-Exos (C_1_: 0.5 mg/mL-0.1 µg/mL; C_2_: 0.5 mg/mL-0.2 µg/mL), SiNPs (C_1_: 25 µg/mL; C_2_: 60 µg/mL), SiNPs-Exos (C_1_: 25 µg/mL- 0.1 µg/mL; C_2_: 60 µg/mL- 0.2 µg/mL), or Exo (C: 0.2 µg/mL) in FCS-free medium and cell viability was measured at indicated time points. Column, mean; bars, ±S.D. *p < 0.05, **** *p* < 0.0001. (**D**) Concept how formation of an (exosomal) corona can result in reduced nanotoxicity. Created with BioRender.com.

As next step, cell viability was determined after treatment with NM-Exo complexes (Fig. 4). To prevent BC formation of serum components, all experiments were performed under serum deprivation. For the first time, we could show that the acquisition of an exosomal corona around Fmoc-Lys NMs could reverse the observed toxic effects on FaDu cells. The reversal of the nanotoxic effect was significant after Fmoc-Lys treatment using high concentrations (1 mg/mL) for at least 24 h (Fig. 4B, C). The observed effect of the exosome-based BC on nanotoxicity was more prominent for the model SiNPs, which exhibit an overall higher toxicity compared to the biocompatible Fmoc-Lys NPs (see also Fig. 3B). Here, cell viability significantly increased from values below 10% for pristine SiNPs to 55–68% for SiNP-Exo complexes. In summary, the formation of an exosomal corona around the NMs significantly reduced their cellular toxicity (Fig. 4D). All results were also confirmed in HeLa cells (Supplementary Fig. S3).

## Conclusion and outlook

The future holds promising prospects for the extensive utilization of nanoparticles (NPs) in diverse fields, including biomedical applications to improve clinical diagnosis and treatment. The effectiveness of NPs is decisively influenced by their physicochemical properties, such as size, biocompatibility, surface chemistry, and adjustable toxicity. Furthermore, NPs designed for the use in biological systems will not keep their initial properties, but will undergo major changes upon contact with biological environments. Here, especially the formation of protein layers, the protein or biomolecule corona (BC), around NPs have been particularly researched in the last decades. Additionally, some studies show that the BC can be also formed on other nano-sized structures, such as exosomes (Exos)^16^. However, after reviewing the current state of research, investigating the role of exosomes in BC formation is a neglected field of research. Here, we aimed to address this novel concept of an exosomal corona formed around NPs.

Numerous studies have been conducted to investigate the ability of NPs to enter the human cells. As a result, it has become important to characterize the physicochemical properties of NPs including their size, shape, surface properties, coating, morphology, surface charge, hydrophobicity, chemical composition, structure and agglomeration state^33^. Formation of BC around EVs has been reported in the literature, and this corona formation may occur universally in biofluids, and corona components may be characteristic for the surrounding matrix. The mechanism behind corona formation here likely involves protein aggregation and electrostatic interactions^17^.

To the best of our knowledge, we here provide first experimental data supporting the hypothesis of exosomal corona formation: Co-incubation of Fmoc-Lys NPs with isolated exosomes resulted in (1) an altered NP surface morphology (shown by TEM) and molecular composition (ATF-FTIR), (2) an increased size and change of zeta potential (DLS), and (3) a reduced nano-toxicity profile (cell viability assays). Control studies conducted with negatively charged SiNPs confirmed these findings, especially for reduced cellular toxicity.

Limitations of our study include the cancer cell background of the isolated exosomes, which may not represent physiological exosome populations. Further studies should also apply exosomes isolated from non-malignant cells, such as mesenchymal stem cells. In future, the detailed content of the exosomal corona has to be profiled by proteomic studies to characterize effects on nanotoxicity, drug delivery capacity, and the immune system. To increase biomedical translation of our in vitro results, comparable studies must be performed in vivo. Of course, there are also several challenges for the practical use of exosome-decorated NPs for biomedical approaches including batch variability, and scalability.

Regarding the fact that Exos are omnipresent in all biological fluids, they play a decisive role in formation of a distinct BC on NPs, and thus have to potential to fundamentally change the surface identity of biological applied NPs. Exos are used as vehicles for targeted cell communication, and bear numerous biological active cargoes. Consequently, decoration of NPs with functional Exos could affect not only the intended function of the NPs, but also their targeting, cellular uptake, and degradation. Here, further studies taking exosomal protein coronas into account are urgently needed. As positive effect, the “native character” of bound Exos may prevent adverse effects and/or clearance of NPs by immune cells. Thus, our results also suggest Exos as extremely promising agents that could help to reduce toxicity in NP-based treatments. Taken together, we want to stimulate the field to take a closer look to the interplay between “artificial” NM systems and “natural” nano-structures, such as exosomes.

## Electronic supplementary material

Below is the link to the electronic supplementary material.

Supplementary Material 1

## Data Availability

The data that support the findings of this study are available from the corresponding authors, [D.G. and M.O], upon reasonable request.
