# Supplementary material for: Synthesis, characterization, and exosomal corona formation of self-assembled dipeptide nanomaterials
Source: Sci Rep. 2025 Apr 19;15:13607. doi: 10.1038/s41598-025-98706-5 (PMC12009354; doi:10.1038/s41598-025-98706-5)
Supplement: Supplementary file 1 — Supplementary Material 1 [file 41598_2025_98706_MOESM1_ESM.docx]

**Supplementary Information**

**Synthesis, characterization, and exosomal corona formation of self-assembled dipeptide nanomaterials**

Burcu Önal Acet^1,2^, Ömür Acet^2,3^, Madita Wandrey^2^, Roland H. Stauber^2^, Désirée Gül^2*^, Mehmet Odabaşı^1*^

*^1^Faculty of Arts and Science, Chemistry Department, Aksaray University, Aksaray, Turkey*

*^2^Department of Otorhinolaryngology Head and Neck Surgery, Molecular and Cellular Oncology, University Medical Center, 55131 Mainz, Germany*

*^3^Vocational School of Health Science, Pharmacy Services Program, Tarsus University, Tarsus, Turkey*

**Figures**

**A B**







**C D**


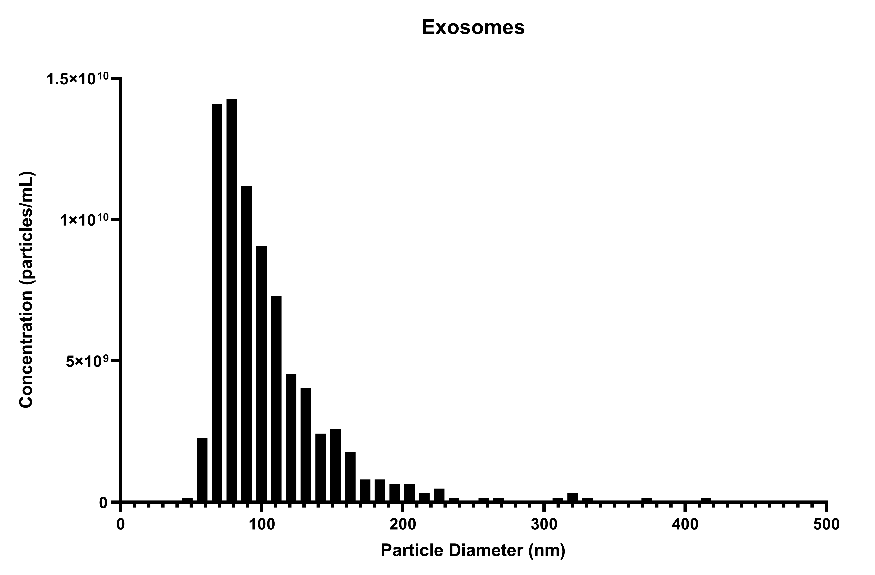

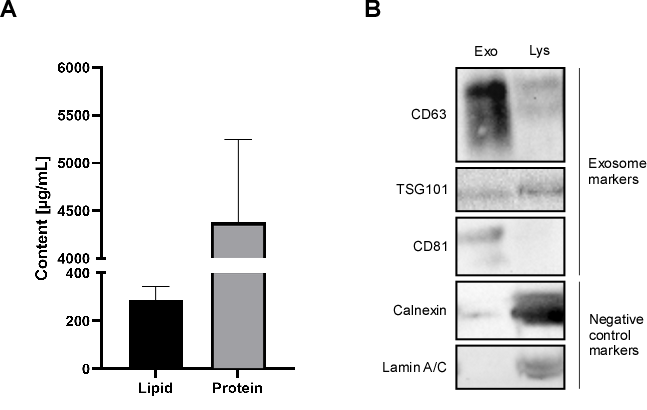


**Figure S1. Characterization of exosomes isolated from cell culture supernatant.** A. and B. Representative cryo-transmission electron microscopy image of isolated exosomes. Scale bars as indicated. C. Representative size distribution of exosomes measured by tunable resistive pulse sensing (TRPS, qNano/ izon). D. Western Blot analysis of exosomes and whole cell lysates for exosomal markers CD63, CD81, and TSG101, as well as negative control markers Calnexin and Lamin A/C.


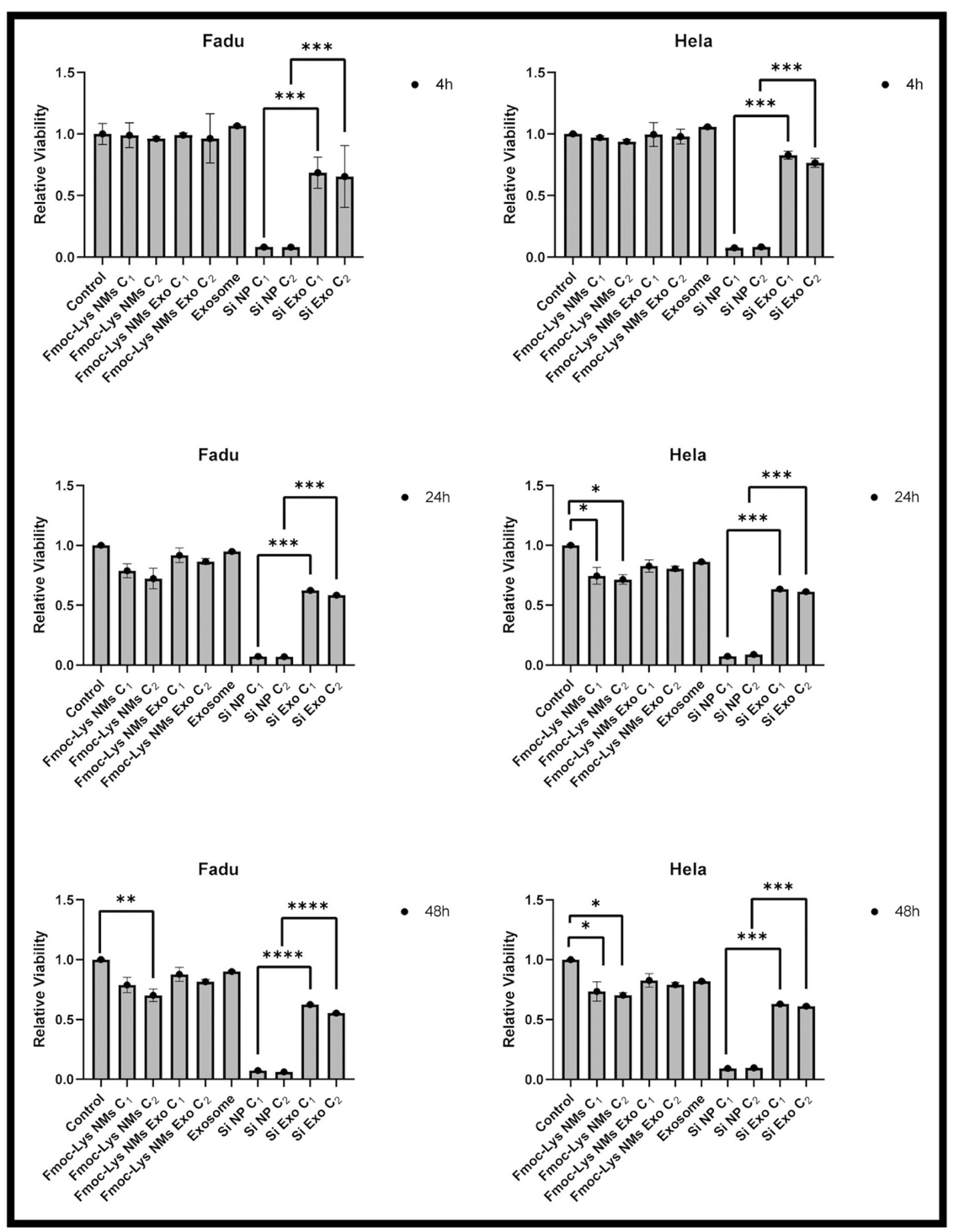


**Figure S2. Acquisition of an exosomal corona on NPs is able to reduce nanotoxicity**. HeLa cells were treated with Fmoc-Lys NMs (C_1_: 0.5 mg/mL; C_2_: 1 mg/mL), Fmoc-Lys NMs-Exos (C_1_: 0.5 mg/mL-0.1 µg/mL; C_2_: 0.5 mg/mL-0.2 µg/mL), SiNPs (C_1_: 25 µg/mL; C_2_: 60 µg/mL), SiNPs-Exos (C_1_: 25 µg/mL- 0.1 µg/mL; C_2_: 60 µg/mL- 0.2 µg/mL), or Exo (C: 0.2 µg/mL) in FCS-free medium and cell viability was measured at indicated time points. Column, mean; bars, ±S.D. *, p <0.05, ****, p <0.0001.
